# Bleaching Teeth by Electricity

**Published:** 1895-04

**Authors:** Albert Westlake

**Affiliations:** Brooklyn, N. Y.


### BLEACHING TEETH BY ELECTRICITY.

BY DR. ALBERT WESTLAKE, BROOKLYN, N. Y.

The cataphoric effect of using pyrozone twenty-five-per-cent. solution in restoring the normal color of teeth in a few minutes is described in the following cases. The same appreciation of current, strength, and resistance must be considered as is used in cataphoresis on live dentine for cocainizing.

The result of continued experiments with ethereal solutions of pyrozone by cataphoresis in bleaching, abscesses, etc., will be embodied in a paper now being prepared on "Electricity in Dental Practice," which will be a supplement to the one read by me at Albany, in May, 1892.

Case No. 1.—Mrs. A. Nervo-bilious temperament; presented right inferior incisor very darkly discolored, a proximal cavity, and incipient abscess. After carefully adjusting rubber involving teeth on both sides, I cleaned the cavity, opened canal, and removed the pulp; I then passed a few shreds of cotton saturated with warm salt water in its place, after which I filled the cavity with pure absorbent cotton saturated with pyrozone twenty-five per-cent. solution ethereal, and applied the positive pole galvanic current, in the shape of a needle, to the moist cotton, and placed the negative pole in the patient's right hand, repeating this three times as the cotton dried.

I commenced with four cells, and increased to about twelve cells, when the tooth began to appear white in patches about the neck and half-way up the crown; this half of the tooth soon presented a bleached condition in sharp contrast to the biting-edge. I then transferred the negative pole and made a short circuit through the upper part of the tooth, after having cut a narrow ridge through the enamel of the biting-edge. I then filled the root-canal and cavity, and the biting-edge with gold. The tooth in other respects still retains a perfectly normal appearance.

This first experiment in cataphoresis for bleaching took place on Friday, March 8. The bleaching did not occupy more than ten minutes. The additional cataphoric effect on the periosteum and adjacent tissue was beneficial, as the tooth is perfectly comfortable at the present writing, three days after the operation.

CASE No. 2.—Miss T. Nervo-sanguine temperament; left superior central incisor had been treated and an attempt made at bleaching the tooth by a dentist in New Jersey. I removed the gold filling, and found that the tooth presented a dark straw-color. I applied the same method of application, but omitted cutting the biting-edge. I found the cavity in this tooth much larger, but as the canal was filled with cement, the resistance was greater, and more current was necessary. I continued the application too long, and secured too great a bleaching effect. I filled the tooth temporarily with gutta-percha, but will blend the extremely-bleached appearance by inserting a lining of cement.

CASE No. 3.—Gentleman. Presenting superior lateral. The method and result were the same as in Case No. 1.

